# Suspended Fœtal Animation

**Published:** 1872-03

**Authors:** A. W. Griggs

**Affiliations:** West-Point, Georgia


### SUSPENDED FŒTAL ANIMATION—(Concluded.)

BY A. W. GRIGGS, M.D., WEST-POINT, GA.

Let us now consider the condition of the fœtus at birth, as what has been said is prefatory, and suggestive mostly of such surroundings as may be expected at the bedside of the obstetric patient, under some of the unfavorable conditions we have mentioned.

The causes of asphyxia may be inherent in the fœtus: from imperfect development of the heart; congenital tuberculosis of the lungs; constitutional frailty arising from disease of the placenta or funis; or from causes which we are unable to appreciate. When a child is born, our apprehensions of danger will be justly awakened should it fail to announce its arrival by the usual signal cry. Our duty is to determine whether animation is *extinct* or *suspended*. In the latter event, prompt measures should be used for its immediate relief. A rapid survey of the circumstances of the labor (which should already have been impressed thoroughly upon the mind) will give direction to correct opinions as to the nature of the difficulty, and general principles will ordinarily indicate the plan to be pursued. If the labor had been protracted, and especially if the liquor amnii had prematurely escaped, or if there was malpresentation, or an unusually large fœtal head, or preternaturally small or distorted pelvis, or if too great an interval had elapsed between the expulsion of the head and that of the trunk, we could but reasonably have expected symptoms denotive of *cerebral congestion*, or apoplexy of authors. I prefer the former title, as it is more clearly expressive of the true condition. There is lividity of the face, and the general surface is purple or blue; the circulation is oppressed, though not intermittent, and there is little or no respiratory effort. All agree to blood-letting by division of the funis as offering the safest and most expeditious means of relief. The application of cold water to the scalp will prove of incalculable service in the case; but if there still be no inspiratory act, inflation of the lungs should be practiced, or some other mode of artificial respiration instituted. I can imagine no condition of asphyxia in which the warm bath is more peremptorily demanded than in this. The child should be immersed up to the neck, and cold cloths constantly applied to the head; the bath-tub should be placed upon a table, so that inflation may be also used by the attendant without the inconvenience and fatigue of the stooping posture, and the temperature of the water should be kept at blood-heat.

Again: suppose that there was hemorrhage, during labor, from placental detachment; a different case will be presented, in which syncope will take the place of cerebral congestion. The fœtus is pallid and relaxed, its condition depending on the amount of blood lost. The child should be laid upon its right side, on a plane inclined at an angle of twenty or twenty-five degrees, the head being lowest; cold water sprinkled in the face; the attendant's fingers dipped in cold water, and applied to the nape of the neck and between the shoulders, or a little cold water dashed upon the epigastrium; and if these fail, Sir Marshall Hall's method, or inflation, should be called to our aid. Such a case occurred in my practice in the county of Coweta, in this State, in 1856, on the plantation of Mr. Nicholas Griffin. The case was that of a negress, in which there was partial placenta prævia and considerable hemorrhage before delivery. The child was born in a state of syncope; and after other means described had failed to establish the respiration fully, I poured half a pint of spirits (whether of whisky or brandy I do not remember) into about a gallon and a half of warm water, in a wash-tub, and placed the child in it, on its back. The vessel was large, and the water did not cover the child by half. I was particular in keeping the water out of its ears. The respiration was soon established. I have used the plan several times in the last sixteen years, satisfactorily, and would recommend the addition of some spirit to the cerebro-spinal bath of Dr. E. T. McDaniel, of Alabama; a gill of brandy to a gallon of warm water will generally be sufficient. Inflation can be practiced while in bath.

Again: asphyxia produced by compression of the placenta or funis umbilicalis will present different symptoms from either of those states to which reference has been made. The fœtus will now be in the condition of one drowned, or rather, smothered; I believe that this is, for the most part, the most favorable case for treatment. Spank the little fellow; apply cold to the occiput, down the cervical and superior dorsal spine; use frictions, artificial respiration, and, if need be, the bath alternately hot and cold. A little blood from the cord might be admissible in some cases, but should be practiced with the greatest care—nor have I known it necessary. Persevere, and you will often be rewarded with success after many hours of toil and anxiety.

Asphyxia is sometimes either caused by or associated with pulmonary congestion. Bleeding at the cord and the use of the warm bath form the basis of the treatment. The exact condition can not, I believe, be well appreciated, unless we take into account the character of the labor. I have seen but *one case*—except in footlings and breech-presentations—and that child cried as soon as the head was expelled; it was at seven months. I turned it upon the right side as soon as it was born, and in a few moments discovered that its respiration was embarrassed, and observed that bloody froth was escaping from its lips. The funis was divided, and about two teaspoonsful of blood taken, and we immersed it in a warm bath, and used frictions after removing it; applied mustard to the spine; wrapped it up in warm flannel. The respiration continued the same—the inspiratory act was short, and the expiratory prolonged, and was accompanied with moaning; the bloody froth also continued; the temperature diminished, and the child died in eight hours.

I have treated several cases—which were in footlings or breech-presentations—in which the same symptoms appeared, and succeeded in restoring the little sufferers upon the plan just indicated. Want of temperature is unfortunate in any case, though it may be overcome; yet, when present in cases of apnœa, associated with congestion or apoplexy of the lungs, it is almost a fatal signal.

I have now related the four principle conditions of suspended fœtal animation as detailed by authors, and as I have found them at the bed-side. I can not, at this time, enter upon a minute description of the various degrees and complications of the condition. I will only allude to cyanosis neanatorum, so philosophically treated by Dr. Charles D. Meigs, of Philadelphia, to whose writings I refer the reader. It is dependent on an admixture of venous with arterial blood, which is effected in the left cardia through the botal foramen, which has not properly closed. The patient is laid on the right side, with the head and trunk elevated at an inclination of fifteen degrees, and prompt relief is insured as long as the position is maintained.

Occasionally we meet with cases of defective fœtal vitality, when we are at sea as to the cause. The respiration will be irregular, and improperly performed. There is an evident want of vigor in the respiratory nerves; the child appears too weak to live; the circulation is still going on through the cord. I have been repeatedly warned against dividing it in these cases, and will not do it at once for fear of injury. Let us consider the fact that aquatic and atmospheric respiration is going on in the same individual at the same time: carbonic acid gas is escaping through the funis, when it should be eliminated by the lungs. Further: we are informed, by the authority of the best physiologists, that the presence of carbonic acid in the blood, circulating about the air-cells, keeps up the respiratory function by reflex action. Now, by compressing the vessels of the funis temporarily, the carbonic acid will seek its natural outlets, and the respiration will improve. If this is found to be the case, ligature the cord and throw the child upon its own resources, and the practice will be successful. I know of no one except myself who has made the experiment; I have done so repeatedly, and have no reason to regret it. Caution is doubtless necessary; it would not do to apply the treatment in the wrong case.

---

Chloride of Ammonium in Hepatitis and Hepatic Abscess. This remsdy is highly lauded in all cases of hepatic derangement where mercury or other resolvents are indicated, both in France and Germany. It is given in doses of one gramme (15 gr.) morning and evening.—*Bull's Therap., Dec.* 15, 1871.

